# Metformin impairs growth of endometrial cancer cells via cell cycle arrest and concomitant autophagy and apoptosis

**DOI:** 10.1186/1475-2867-14-53

**Published:** 2014-06-16

**Authors:** Akimasa Takahashi, Fuminori Kimura, Akiyoshi Yamanaka, Akie Takebayashi, Nobuyuki Kita, Kentaro Takahashi, Takashi Murakami

**Affiliations:** 1 Department of Obstetrics and Gynecology, Shiga University of Medical Science, Seta-Tsukinowa-cho, Otsu, Shiga 520-2192, Japan

**Keywords:** Metformin, Endometrial cancer, Autophagy, Apoptosis

## Abstract

**Background:**

Effective therapies for early endometrial cancer usually involve surgical excision and consequent infertility Therefore, new treatment approaches that preserve fertility should be developed. Metformin, a well-tolerated anti-diabetic drug, can inhibit cancer cell growth. However, the mechanism of metformin action is not well understood. Here we investigate the roles of autophagy and apoptosis in the anti-cancer effects of metformin on endometrial cancer cells.

**Methods:**

Ishikawa endometrial cancer cells were treated with metformin. WST-8 assays, colony formation assays, flow cytometry, caspase luminescence measurement, immunofluorescence, and western blots were used to assess the effects of metformin on cell viability, proliferation, cell cycle progression, apoptosis, and autophagy.

**Results:**

Metformin-treated cells exhibited significantly lower viability and proliferation and significantly more cell cycle arrest in G1 and G2/M than control cells. These cells also exhibited significantly more apoptosis via both intrinsic and extrinsic pathways. In addition, metformin treatment induced autophagy. Inhibition of autophagy, either by Beclin1 knockdown or by 3-methyladenine-mediated inhibition of caspase-3/7, suppressed the anti-proliferative effects of metformin on endometrial cancer cells. These findings indicate that the anti-proliferative effects and apoptosis caused by metformin are partially or completely dependent on autophagy.

**Conclusions:**

We showed that metformin suppresses endometrial cancer cell growth via cell cycle arrest and concomitant autophagy and apoptosis.

## Background

Endometrial cancer is one of the most common gynecologic malignancies in the United States [1], and its incidence is rapidly increasing in Japan [2]. Approximately 80% of endometrial cancers are diagnosed at an early stage and are completely cured with hysterectomy [3,4]. In addition, approximately 25% of all cases are diagnosed in premenopausal women, and 3%–14% of all cases are diagnosed before 40 years of age [5-8]. Endometrial cancer in young women poses a therapeutic dilemma because preservation of fertility is often a major concern. Progesterone and medroxyprogesterone acetate are often used to treat endometrial cancers in patients who desire to preserve their fertility [9]. Some younger women with endometrial cancer present with obesity, irregular menses, chronic anovulation, polycystic ovarian syndrome, insulin resistance, type 2 diabetes mellitus, or a combination [7,10]. Elimination of such conditions using low-dose cyclic progestin may decrease recurrence or *de novo* development of endometrial cancer. However, maintenance treatment with progestin prohibits pregnancy, and the therapeutic effect of progestin in endometrial cancers appears to be inadequate. Therefore, new approaches to the treatment and prevention of endometrial cancer must be developed for women trying to conceive.

The biguanide drug metformin is among the most prescribed drug for the treatment of type 2 diabetes worldwide. Metformin (1,1-dimethylbiguanide hydrochloride) is a well-tolerated drug that has numerous cellular effects in multiple tissues. The main anti-hyperglycemic effect is believed to be due to the suppression of hepatic glucose production [11]. In addition, metformin has been reported to inhibit the growth of various cancers [12-18], including endometrial cancer [19]. Metformin activates AMPK, a critical cellular energy sensor. Activation of AMPK suppresses the mTOR; this cascade leads to reduced protein synthesis and cell proliferation [20]. In addition, higher doses of metformin (2–5 mM) reportedly induce apoptosis in endometrial cancer cell lines [20]. Whether metformin induces other forms of cell death such as autophagy is unknown.

Programmed cell death refers to any type of cell death mediated by an intracellular program [21]. Apoptosis is type-I programmed cell death, which is morphologically characterized by cell shrinkage, chromatin condensation, nuclear fragmentation, and formation of apoptotic bodies. Autophagic cell death is type-II programmed cell death, which is characterized by the accumulation of multi-lamellar vesicles that engulf the cytoplasm and organelles [22]. Apoptosis has long been known to play an important role in the response to several chemotherapeutic agents; however, the importance of treatment-induced autophagic cell death in tumor regression has only recently been recognized [23,24]. Metformin induces apoptosis in some cancers [12,14,25] and autophagy in other, including melanoma, lymphoma, and colon cancer [12,17,18]. Multiple functional relationships between apoptosis and autophagy in cancer cells have been reported. Thus, a better understanding of the interactions between apoptosis and autophagy may be a key to continued improvement of cancer treatments.

Here we used an endometrial cancer cell line to investigate the anti-cancer activity of metformin. We focused on the role of autophagy and its effects on apoptotic cell death.

## Methods

### Reagents and antibodies

Metformin (1,1-dimethylbiguanide hydrochloride), 3-methyladenine (3MA), chloroquine (CQ), and siRNA were purchased from Sigma Aldrich (St. Louis, MI, USA). Anti-actin antibody was purchased from Sigma; all other antibodies were purchased from Cell Signaling Technology (Beverly, MA, USA). Modified Eagle’s medium (MEM), non-essential amino acids (NEAA), and trypsin/EDTA (0.25% trypsin, 1 mM EDTA) were purchased from Wako Pure Chemical Industries (Osaka, Japan). Antibiotics/antimycotics (ABAM) were purchased from Gibco (Carlsbad, CA, USA). Cell counting kit-8 (CCK-8) was purchased from Dojindo Laboratories (Tokyo, Japan). Caspase-Glo assay kits were purchased from Promega (Madison, WI, USA). FITC Annexin V apoptosis detection kit I, FITC BrdU Flow Kit, and BD MitoScreen (JC-1) were purchased from BD Pharmingen (San Diego, CA, USA). Acridine orange (AO) was purchased from Molecular Probes (Eugene, OR, USA). Lipofectamine 2000 was purchased from Invitrogen (Carlsbad, CA, USA).

### Cell culture, cell viability assay, and colony formation assay

The Ishikawa human endometrial adenocarcinoma cell line was purchased from the European Collection of Cell Culture (ECACC, Salisbury, UK). Ishikawa cells were cultured in MEM supplemented with l-glutamine (2 mM), 5% (v/v) FBS, 1% NEAA, and ABAM at 37°C in a humidified atmosphere with 5% CO_2_.

We performed this work by using only cell line, but not clinical samples. Therefore, this work has been granted exemption from the Ethics Committee of Shiga University of Medical Science.

The WST-8 assay was used to measure cell viability. Cells were plated on 96-well plates at a density of 1 × 10^4^ cells/well in 100 μL medium. At 24 h after seeding, metformin (0, 0.01, 1, 5, 10, or 20 mM) was added to each well and cells were cultured for an additional 48 h. CCK-8 solution (10 μL) was then added to each well, and the plates were incubated at 37°C for 2 h. The absorbance of WST-8 formazan was measured at 450 nm using a microplate reader.

To measure colony formation, adherent Ishikawa cells were trypsinized and 1000 viable cells (depending on the experiment) were subcultured in 60-mm plates; each treatment was tested in triplicate. After 24 h, the medium was replaced with fresh culture medium containing metformin (0, 0.01, 1, 5, 10, or 20 mM) in a 37°C humidified atmosphere with 95% air and 5% CO_2_ and grown for 2 weeks. The culture medium was replaced every 3 days. Cell clones were stained for 15 min with a solution containing 0.5% crystal violet and 25% methanol in water. Stained cells were rinsed three times with tap water to remove excess dye. Each dish was then washed and dried, and the number of colonies/plate was macroscopically counted. Colonies were defined as those containing >50 cells by microscopic examination.

### Assessment of cell cycle, apoptosis, and mitochondrial membrane potential via flow cytometry

To assess cell cycle progression, cells were seeded onto 60-mm plates and incubated for 24 h to allow for exponential growth. Ishikawa cells were incubated with or without metformin for an additional 48 h. All cells were incubated with 10 μM BrdU (BD Pharmingen) for 30 min; BrdU-labeled cells were then harvested, fixed, permeabilized, and stained with FITC-conjugated anti-BrdU antibody and 7-AAD, according to the manufacturer’s instructions. A flow cytometer (BD, FACSCalibur, San Jose, CA, USA) was used to assess DNA content and cell cycle phase.

Annexin V-FITC apoptosis detection kits were used according to the manufacturer’s instructions to measure apoptosis. Cells were incubated with or without metformin for 48 h, collected and washed with PBS, gently resuspended in annexin V binding buffer, and incubated with annexin V-FITC/7-AAD. Flow cytometry was performed using CellQuest Pro software (BD).

A mitochondrial membrane potential detection kit was used according to the manufacturer’s instructions to measure mitochondrial membrane potential (*Δψm*). In brief, cells were treated with or without metformin, resuspended in 0.5 mL of JC-1 solution, and incubated at 37°C for 15 min. Cells were then rinsed before flow cytometry. A dot plot of red (living cells with intact *Δψm*) versus green fluorescence (cells lacking *Δψm*) was generated. Data were expressed as the percentage of cells with intact *Δψm*.

### Caspase activity

The Caspase-Glo™ 3/7, Caspase-Glo™ 8 or Caspase-Glo™ 9 assay kit was used according to the manufacturer’s instructions to measure the activity of caspase 3/7, caspase-8 or caspase-9, respectively. In brief, 50 μL of cell lysate (cytosolic extracts, 20 μg) was incubated in 50 μL of Caspase-Glo reagent at room temperature for 1 h. After incubation, the luminescence of each sample was measured in a plate-reading luminometer (Tecan, Linz, Austria).

### Detection and quantification of autophagic cells by staining with acridine orange

To identify autophagic cells, the volume of the cellular acidic compartment was visualized by AO staining [26]. Cells were seeded in 60-mm culture dishes and treated as described above. After 48 h of treatment with or without metformin, cells were incubated with medium containing 5 μg/mL AO for 15 min. The AO medium was then removed, cells were washed once with PBS, and fresh medium was added. Fluorescence micrographs were taken using an Olympus inverted fluorescence microscope (Olympus Corp., Tokyo, Japan). All images presented are at the same magnification. Flow cytometry was used to determine the number of cells with acidic vesicular organelles (AVOs). Cells were trypsinized and harvested; BD FACSCalibur and BD CellQuest Pro software was used to analyze the cells. A minimum of 10,000 cells within the gated region was analyzed for each treatment.

### RNA interference

Lipofectamine 2000 reagent and the Invitrogen protocol were used to introduce Beclin-1 siRNA (Sigma-Aldrich; seq1 #SASI_Hs02_00336256) or a scramble control siRNA sequence (Sigma-Aldrich; #SIC001) into Ishikawa cells. Cells were then incubated for 48 h prior to metformin treatment (0, 5, or 10 mM).

### Western blot analysis

Ishikawa cells (2 × 10^6^/dish) were seeded in 100-mm culture dishes and cultured for 24 h. After metformin treatment, cells were lysed in RIPA lysis buffer containing a protease-inhibitor cocktail (“Complete” protease inhibitor mixture; Roche Applied Science, Indianapolis, IN) on ice for 30 min. Suspensions of lysed cells were centrifuged at 14 000 × *g* at 4°C for 10 min; supernatants containing soluble cellular proteins were collected and stored at -80°C until use. BCA protein assay kits were used to measure protein concentration. Furthermore, 15 μg of protein was resuspended in sample buffer and separated on a 4%–20% tris-glycine gradient gel using the SDS-PAGE system. Resolved proteins were transferred to PVDF membrane, which was blocked with 5% milk in tris-buffered saline/0.1% Tween 20. Immunodetection was performed using each primary antibody. The membranes were incubated with donkey anti-rabbit horseradish peroxidase (HRP)-conjugated secondary antibody (1:5000 dilution). The ECL Western Blotting Detection System (GE Healthcare, Little Chalfont, UK) was used to detect signals, which were visualized using a LAS-4000 mini (GE Healthcare). Actin was used as the loading control.

### Statistical analysis

All data points represent the mean of at least three independent measurements and are expressed as the mean ± standard deviation. SPSS ver. 20 was used to perform one-way ANOVA and Tukey’s *post hoc* test or Student’s *t*-test, as appropriate. A significance threshold of p < 0.05 was used.

## Results

### Metformin inhibits growth of Ishikawa endometrial cancer cells

**Figure 1:**
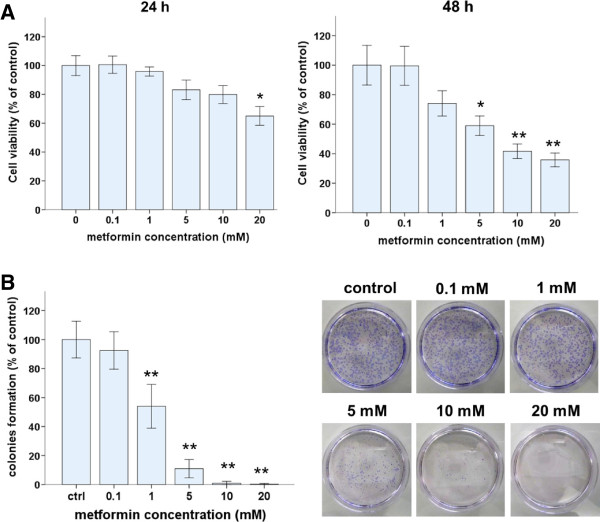
**Metformin inhibits Ishikawa cell growth and induces cell death. (A)** WST-8 assays were used to measure the viability of Ishikawa cells after treatment with metformin (0, 0.1, 1, 5, 10, or 20 mM) for 24 or 48 h. **(B)** Colony formation assays were used to measure the colonogenicity of Ishikawa cells after metformin treatment. The number of untreated cells was set as 100%. The data are mean values from triplicate experiments. *p < 0.05, **p < 0.01, one way ANOVA, post hoc comparisons, Tukey’s test. Columns, mean; error bars, SD.

WST-8 and colony formation assays were used to assess the effects of metformin on the viability of Ishikawa endometrial cancer cells. The number of viable cells decreased with increasing concentrations of metformin for 24- or 48-h treatments (Figure 1A). After 24 h, 20 mM of metformin significantly reduced the number of viable cells but 0.01–10 mM metformin did not. After 48 h, metformin at 5 mM or more significantly reduced the number of viable cells. At 48 h, IC50 of metformin was 6.78 mM.

The ability of metformin-treated and control Ishikawa cells to form colonies on 60-mm culture plates within two weeks was examined. Metformin at concentrations as low as 1 mM, significantly reduced colony formation (Figure 1B), and the inhibitory effect of metformin on colony formation was dose dependent. Metformin at 5 mM or more reduced colony formation to 10% of that of untreated control cells. Based on these results and those in several published reports [13,17,27,28], 5 or 10 mM metformin was used in the following experiments.

### Metformin induces cell cycle arrest and modulates cell cycle proteins in Ishikawa endometrial cancer cells

**Figure 2:**
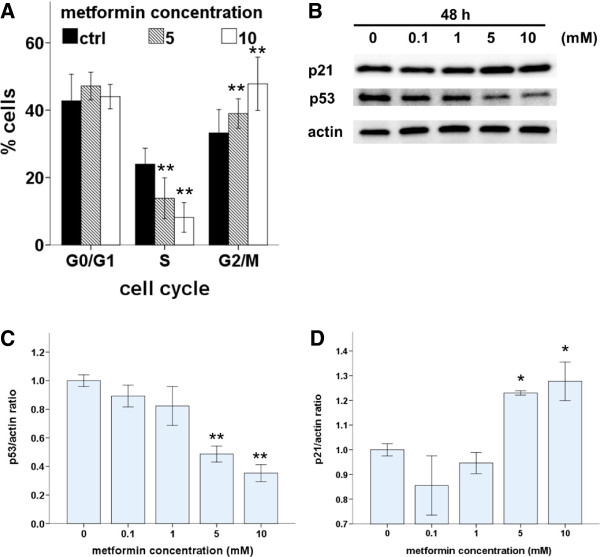
**Metformin induces cell cycle arrest. (A)** Ishikawa cells were treated with metformin (0, 5, or 10 mM) for 48 h. After fixation, the cell cycle was analyzed by flow cytometry. Quantitative analysis of percentage gated cells at the G0/G1, S, and G2/M phases are shown. Data are shown as mean ± SD of three independent replicate measurements. *p < 0.05 and **p < 0.01 vs. untreated cells. **(B)** Ishikawa cells were treated with metformin (0, 0.1, 1, 5, or 10 mM) for 48 h. Cell lysates were separated by SDS-PAGE and analyzed on western blots with the indicated antibodies. Actin was used as a loading control. One representative experiment of three experiments is shown. **(C and D)** Densitometric quantitation of p21/actin and p53/actin protein expression levels is shown as fold changes. One representative experiment of three is shown. *p < 0.05 and **p < 0.01 vs. untreated cells.

To investigate the underlying mechanisms of metformin-induced growth inhibition in Ishikawa cells, we first evaluated the effect of metformin on cell proliferation and cell-cycle progression. Cell-cycle profiles were analyzed after 48 h of metformin treatment. There were significantly fewer S-phase cells and significantly more G2/M cells in metformin-treated cultures compared with those in control cultures, and these effects were dose dependent (Figure 2A). Furthermore, we used western blots to assess the effects of metformin on the expression of two cell cycle regulators, p53 and p21 (Figure 2B). Expression of p53 decreased in a dose-dependent manner with metformin treatment (Figure 2C). The induction of p21, a cell-cycle blocker, increased in a dose-dependent manner with metformin treatment (Figure 2D). These results indicate that metformin induced p21 expression, which led to cell cycle arrest in G1 and G2/M via a p53-independent pathway.

### Metformin induces apoptosis of Ishikawa endometrial cancer cells via intrinsic and extrinsic pathways

**Figure 3:**
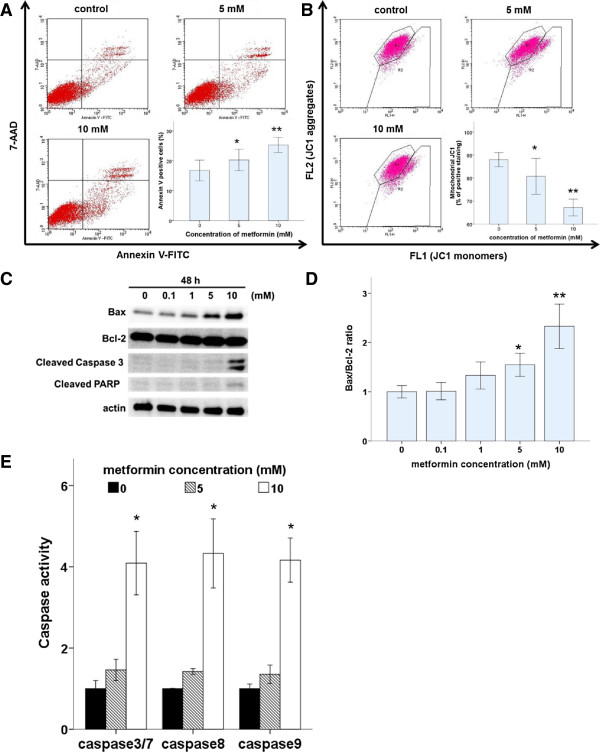
**Metformin induces apoptosis in Ishikawa cells. (A)** Ishikawa cells were treated with metformin (0, 5, or 10 mM) for 48 h. Cells were harvested and stained with annexin V-FITC and 7-AAD, and cell apoptosis was analyzed using flow cytometry. *p < 0.05 and **p < 0.01 vs. untreated cells. **(B)** Ishikawa cells were treated with metformin (0, 5, or 10 mM) for 48 h, stained with JC-1, incubated, and analyzed using flow cytometry. *p < 0.05 and **p < 0.01 vs. untreated cells. (**C** and **D**) Ishikawa cells were treated with metformin (0, 0.1, 1, 5, or 10 mM) for 48 h. Cell lysates were separated by SDS-PAGE and analyzed on western blots probed with the indicated antibodies. Actin was used as a loading control. Densitometric quantitation of Bax/Bcl-2 protein expression ratios are shown as fold changes. One representative experiment of three is shown. **(E)** Ishikawa cells were incubated with metformin (0, 5, or 10 mM) for 48 h, followed by assays of caspase-3/7, -8, and -9 activity. *p < 0.05 and **p < 0.01 vs. untreated cells.

To assess whether the induction of apoptosis also contributed to metformin-mediated inhibition of Ishikawa cell growth, the proportion of apoptotic cells was measured. After cells were incubated with or without metformin (5 or 10 mM) for 48 h, the proportion of apoptotic cells was measured by flow cytometric of annexin V expression and JC-1 staining, which indicates the presence of a mitochondrial membrane potential (Figure 3A and 3B). Our results demonstrate that the proportion of apoptotic cells was higher in metformin-treated cultures compared with that in controls.To understand the mechanism by which metformin induced apoptosis in Ishikawa cells, we examined pro-apoptotic activity. Apoptosis can be activated through two main pathways: the intrinsic mitochondria-dependent pathway and the extrinsic death-receptor-dependent pathway. Caspase-8 is predominantly activated by signals from the extrinsic death-receptor pathway, while caspase-9 activation is dependent primarily on the intrinsic mitochondrial pathway. Together, pro-apoptotic Bax and anti-apoptotic Bcl-2 play an important role in mitochondrial outer membrane permeabilization. Metformin treatment induced a marked, dose-dependent increase in the Bax/Bcl-2 ratio (Figure 3D). Furthermore, metformin-mediated apoptotic death was accompanied by the activation of caspase, which is the principal apoptosis-executing enzyme. Fluorescence calorimetric analysis demonstrated that metformin treatment induced the activation of caspase-3/7, -8, and -9 (Figure 3E). Consistent with the induction of apoptosis, western blots revealed that metformin treatment led to cleavage of caspase-3 and PARP in Ishikawa cells in a dose-dependent manner (Figure 3C).

### Metformin triggers autophagy in Ishikawa cells

**Figure 4:**
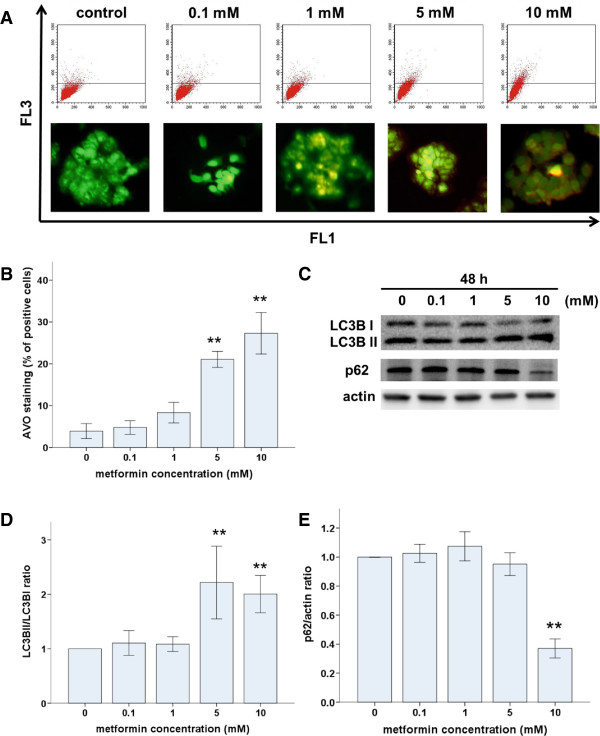
**Metformin triggers autophagy in Ishikawa cells. (A)** Ishikawa cells were incubated with metformin (0, 0.1, 1, 5, or 10 mM) for 48 h. The presence of acridine orange-stained intracellular vesicles was demonstrated by fluorescent microscopy (lower panel) and flow cytometry, showing an increase in red fluorescence (FL3) intensity (upper panel). **(B)** Autophagy was quantified by measuring the percentage of cells with bright red fluorescence (FL3-positive cells; data are presented as the mean ± SD of three independent measurements, *p < 0.05). **(C)** Immunoblot analysis of LC3 conversion and p62 levels in Ishikawa cells treated with metformin (0, 0.1, 1, 5, or 10 mM). (**D** and **E**) Densitometric quantitation of LC3B-II/LC3B-I and p62/actin protein expression ratios are shown as fold changes. *p < 0.05 and **p < 0.01 vs. untreated cells.

To determine whether metformin induced autophagy in Ishikawa cells, we used AO to stain AVOs, including autophagic vacuoles. Untreated Ishikawa cells exhibited bright green fluorescence in the cytoplasm and nuclei and lacked bright red fluorescence. In contrast, metformin-treated cells exhibited AVOs, identified as bright red compartments (Figure 4A). The number of AVOs was significantly higher in metformin-treated cells compared with that in untreated controls, and this effect was dose dependent (Figure 4A). Levels of LC3B (an autophagosome component) and p62 (an autophagosome target) positively and negatively correlate with autophagy, respectively. Therefore, we used western blots to assess LC3B-I to LC3B-II conversion and p62 protein levels. As expected, metformin treatment induced significant LC3 I to II conversion (Figure 4C and D) and a decrease in p62 levels (Figure 4C and E) in a dose-dependent manner. Taken together, these results demonstrate that metformin induced autophagy in Ishikawa cells.

### Inhibition of autophagy reduced metformin-induced apoptosis in Ishikawa cells

**Figure 5:**
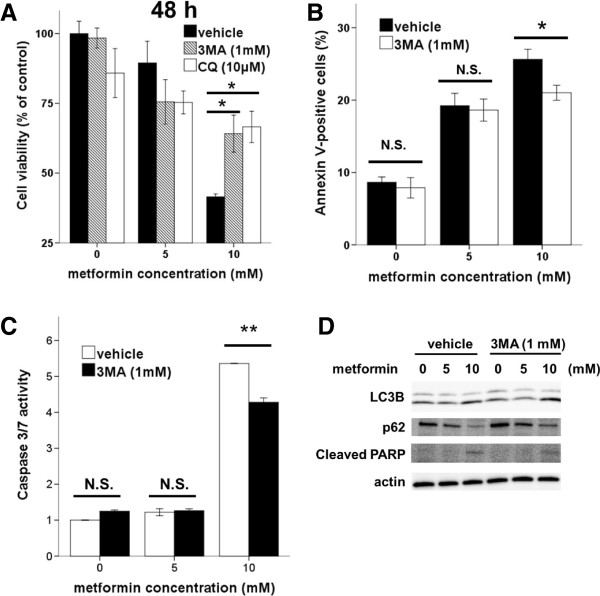
**Pharmacologic inhibition of autophagy reduces metformin-mediated apoptotic cell death. (A)** Ishikawa cells were seeded in 96-well plates and incubated with the indicated dose of metformin (with or without 3MA or CQ) for 48 h; cell viability was determined by WST-8 assays. Data are presented as the mean ± SD of three independent replicate measurements. ^*^p < 0.05 and ^**^p < 0.01 vs. 3MA or CQ untreated cells. **(B)** Flow cytometry of apoptosis in Ishikawa cells treated with metformin (with or without 3MA) for 48 h. ^*^p < 0.05 and ^**^p < 0.01 vs. 3MA untreated cells. **(C)** Ishikawa cells were incubated with the indicated dose of metformin alone or combined with 3MA for 48 h. Cells were then lysed to measure caspase-3/7 activity. *p < 0.05 and **p < 0.01 vs. 3MA untreated cells. **(D)** Ishikawa cells were treated with the indicated dose of metformin (±3MA) for 48 h, and cell lysates were subjected to western blot analysis using antibodies against LC3B, p62, and cleaved PARP. Actin was used as a loading control.

**Figure 6:**
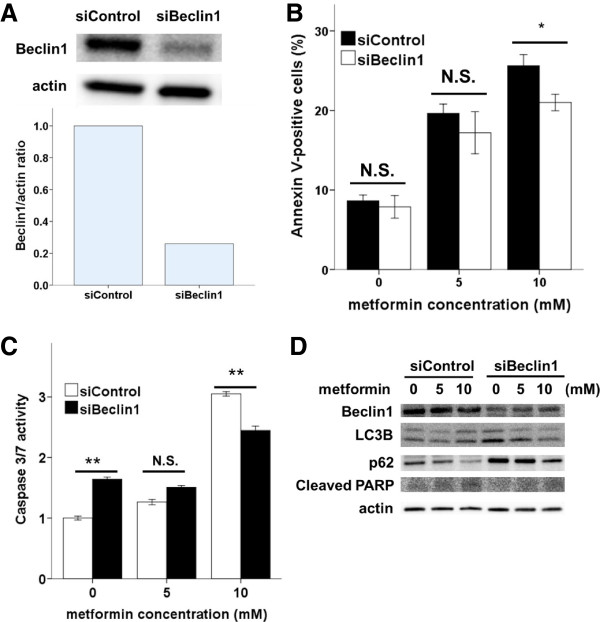
**Genetic inhibition of autophagy reduces metformin-mediated apoptotic cell death. (A)** Western blot analysis of Beclin1 expression in siBeclin1 and control Ishikawa cells. **(B)** Beclin1 expression was blocked in Ishikawa cells by Beclin1 siRNA; cells were incubated with the indicated concentrations of metformin for 48 h. Cells were then harvested and analyzed by flow cytometry. *p < 0.05 and **p < 0.01 vs. Control siRNA cells. **(C)** Beclin1 siRNA Ishikawa cells were incubated with the indicated concentrations of metformin for 48 h. Cells were then lysed to measure caspase-3/7 activity. *p < 0.05 and **p < 0.01 vs. Control siRNA cells. **(D)** Beclin1, LC3B, p62, and cleaved PARP expression were examined in siBeclin1-treated and control cells. Actin expression was used as a loading control.

To determine the relationship between apoptosis and autophagy in Ishikawa cells, we inhibited autophagy either pharmacologically (via 3MA or CQ) or genetically, and assessed the effects on metformin-mediated apoptosis. A WST-8 assay showed that 3MA and CQ treatment significantly enhanced the viability of metformin-treated (10 mM) cells (Figure 5A). On addition, flow cytometric analysis showed that 3MA treatment caused a marked decrease in the proportion of metformin-treated (10 mM) apoptotic cells (Figure 5B). Moreover, 3MA treatment caused a significant reduction in caspase activity in metformin-treated (10 mM) cells (Figure 5C). Thus, these findings revealed that inhibition of metformin-mediated autophagy reduced apoptosis in Ishikawa cells.To confirm these results, we used siRNA to repress expression of the autophagy regulator Beclin1 in Ishikawa cells. Beclin1 siRNA knocked down Beclin1 expression by approximately 75% (Figure 6A). Upon metformin treatment, significantly fewer Annexin-V-positive cells were observed in Beclin1siRNA cells compared with that in controls (Figure 6B). The inhibition of autophagy by Beclin1 siRNA resulted in decreases in caspase-3/7 activity (Figure 6C), PARP cleavage, and LC3-II and increases in p62 (Figure 6D), as did pharmacologic inhibition of autophagy by 3MA (Figure 5D). These results demonstrate that the inhibition of autophagy reduced apoptosis associated with metformin treatment.

## Discussion

Recent data indicate that metformin may be a useful anti-proliferation agent for some types of cancer. The potential role of metformin in treating endometrial cancer has been explored in a number of *in vitro* studies [19,29,30]. However, the anti-tumor effects of metformin are not completely understood. Furthermore, the effect of metformin on autophagy has not been investigated in endometrial cancer cells. Here we demonstrate that metformin induced caspase-dependent apoptosis and suppressed proliferation by upregulating the cyclin-dependent kinase inhibitor p21 and inducing both G1 and G2/M arrest. In addition, we revealed that metformin promoted the formation of AVOs, the conversion of LC3-I to LC3-II, and the degradation of p62. Moreover, both pharmaco logic and genetic inhibition of autophagy reduced metformin-induced apoptosis. To the best of our knowledge, this is the first report to demonstrate that metformin induces autophagy and that autophagy and apoptosis are linked processes.

Several studies have indicated that metformin treatment decreases cancer cell viability by inducing apoptosis. Cantrell et al. showed that metformin increased activation of caspase-3 in human endometrial cancer cells in a dose-dependent manner [19]. Hanna et al. suggested that metformin induces apoptosis [20]. Similar to the results of these studies, we observed that metformin treatment of Ishikawa endometrial cancer cells induces a significant increase in apoptosis in a dose-dependent manner.

To elucidate the mechanism of metformin-induced apoptosis, we investigated mitochondrial function and caspase activity in Ishikawa cells. We observed that metformin treatment altered the expression of Bcl-2 family proteins, PARP cleavage, and the activation of caspase-3/7, -8, and -9. Caspase-8 is essential for death-receptor-mediated apoptosis, while caspase-9 is essential for mitochondria-mediated apoptosis. These 2 pathways converge on caspase-3/7 activation, leading to subsequent activation of other caspases. Our results are similar to those of previous findings demonstrating that metformin induces significant increases in apoptosis in pancreatic cell lines and that metformin-induced apoptosis is associated with PARP cleavage, which is dependent on activation of caspase-3, -8, and -9 [14]. Thus, metformin may modulate apoptotic cell death via extrinsic and intrinsic pathways in Ishikawa cells.

In addition, metformin has been shown to induce arrest of the cell cycle in cancer cell lines [31]. Cantrell et al. showed that metformin induces G0/G1 cell cycle arrest in Ishikawa cells [19]. However, we observed that metformin blocked cell cycle progression not only in G0/G1 but also in the G2/M phase. This apparent discrepancy may result from differences in incubation time, pharmacologic dose or both. G0/G1 cell cycle arrest resulted from a 24-h incubation [19], and G0/G1 and G2/M phase arrest resulted from a 48-h incubation. These findings suggest that metformin may block the cell cycle at two points. We observed that the cyclin-dependent kinase inhibitor p21, which plays an important role in cell-cycle arrest, was activated by metformin. Notably, *p21* is among the genes most consistently induced by metformin [32]. Recent reports indicate that p21 is not only a well-established negative regulator of the G1/S transition but also an inhibitor of the CDK1/cyclin B complex that maintains G2/M arrest [33,34]. These reports support our supposition that the G2/M phase cell cycle block occurs at 48 h.

Alternatively, it is possible that low doses of metformin cause G0/G1 arrest, whereas higher doses cause G2/M arrest. High metformin concentrations induce more p21 expression; therefore, they may induce apoptosis of cells not only in G0/G1 but also in the G2/M cell cycle arrest. Moreover, p21 expression is induced by both p53-dependent and -independent mechanisms. Mutations in the p53 gene are reportedly evident in >50% of all known cancer types. These mutations are recognized as one of the major events in carcinogenesis, and the Ishikawa cell line also has a p53 mutation [35]. Therefore, agents that induce p21 expression through a p53-independent pathway may have potential as candidate drugs. Histone deacetylase (HDAC) inhibitors, such as Psammaplin A, suppress cell proliferation and induce apoptosis in Ishikawa cells via p53-independent upregulation of p21 expression [36]. Our results indicate that metformin treatment of Ishikawa cells increased p21 expression but also decreased mutant p53 expression. These findings also indicate that metformin-induced p21 expression may be regulated through a p53-independent mechanism. Therefore, we propose that metformin induces cell-cycle arrest in Ishikawa endometrial cancer cells both at G0/G1 and G2/M by activating p21 via a p53-independent pathway.

Autophagy is a process where the cytosol and organelles become encased in vacuoles called autophagosomes. Although autophagy is primarily a protective process for the cell, it can play a role in cell death [37]. Therefore, autophagy is considered to be a double-edged sword. A recent work highlights the prosurvival role of autophagy in cancer cells [38]. Alternatively, autophagy may confer a disadvantage on cancer cells [12]. The variability in the effects of autophagy on cancer cells may depend on the cell type, cell cycle phase, genetic background, and microenvironment [39]. When the autophagic capacity of cancer cells is reached, apoptosis is promoted [40]. This finding is particularly interesting because metformin can induce autophagy in colon cancer and melanoma [12,17], as well as Ishikawa endometrial cancer cells, as demonstrated here.

Metformin induced apoptosis and autophagy in Ishikawa endometrial cells. Because autophagy has been implicated in the promotion and inhibition of cell survival [21], we were interested in the role of autophagy in metformin-mediated apoptosis. To determine whether the processes of autophagy and apoptosis are linked, we performed several experiments following the inhibition or induction of autophagy. We observed that both pharmacologic and genetic inhibition of autophagy promoted cancer cell survival and reduced metformin-induced apoptosis. In addition, our results show that inhibition of autophagy decreased the cleavage of PARP (Figure 5D and 6D) and the activation of caspase-3/7, -8, and -9 (data not shown). These findings indicate that inhibitors of autophagy enhanced both intrinsic and extrinsic activation of apoptosis. Taken together, these data suggest that metformin induces autophagic cell death in Ishikawa endometrial cancer cells. To the best of our knowledge, this is the first demonstration that metformin promotes the elimination of endometrial cancer cells through concomitant regulation of autophagy and apoptosis. These results are based on *in vitro* studies only, and further *in vivo* studies are necessary.

## Conclusions

We demonstrate that metformin is cytotoxic to Ishikawa endometrial cancer cells. Several mechanisms underlying the anti-tumor effects of metformin in Ishikawa cells are revealed by the data presented here. Metformin was shown to inhibit Ishikawa endometrial cancer cell proliferation through the induction of cell cycle arrest and caspase-dependent apoptosis and enhanced autophagic flux. In addition, we showed that pharmacological or genetic inhibition of autophagy decreased metformin-induced apoptotic cell death. These observations indicate that metformin may be a promising agent for the treatment of early endometrial cancer. In addition, our findings may provide insight into the role of autophagy in anti-cancer therapies.

## Abbreviations

AMPK: Adenosine-monophosphate-activated protein kinase; mTOR: Mammalian target of rapamycin; FITC: Fluorescein isothiocyanate; WST-8 assay: Modified 3-(4,5-dimethylthiazol-2-yl)-2,5-diphenyltetrazolium assay; 7-AAD: 7-Amino-Actinomycin D; FACS: Fluorescence activated cell sorting; AO: Acridine orange; AVOs: Acidic vesicular organelles; RIPA: Radioimmunoprecipitation assay buffer; PARP: poly (ADP-ribose) polymerase; CDK1: Cyclin-dependent kinase 1.

## Competing interests

The authors declare that they have no conflicts of interest concerning the work presented here.

## Authors’ contributions

AT designed and performed research, analyzed data, and wrote manuscript; AT and AY assisted with flow cytomety analysis; FK assisted in witing and editing paper; NK, KT and TM assisted in research design, oversaw data analysis. All authors read and approved the final manuscript.
